# Vitamin D status and obesity in children from Chile

**DOI:** 10.1038/s41430-021-01043-9

**Published:** 2021-11-12

**Authors:** Francisco Pérez-Bravo, Lissette Duarte, Miguel Arredondo-Olguín, Germán Iñiguez, Oscar Castillo-Valenzuela

**Affiliations:** 1 Institute of Nutrition and Food Technology (INTA), University of Chile, Santiago, Chile; 2 Nutrigenomics Laboratory, Nutrition Department, Faculty of Medicine, University of Chile, Santiago, Chile; 3 Nutrition and Dietetics School, Universidad Finis Terrae, Santiago, Chile; 4 Maternal and Child Research Institute (IDIMI), School of Medicine, University of Chile, Santiago, Chile

**Keywords:** Obesity, Nutrition

## Abstract

**Background:**

Vitamin D [25(OH)D] is essential for normal bone development and maintenance. Furthermore, its deficiency has been associated with obesity, cardiovascular diseases, insulin resistance, autoimmune diseases, and certain cancers.

**Objective:**

To determine the incidence of serum 25(OH)D deficiency (<20 ng/ml) among apparently healthy Chilean children (4–14 years old) from three Chilean geographic areas during May–September 2018.

**Materials and methods:**

Serum 25(OH)D levels were measured by a competitive protein-binding ELISA assay in 1134 children, and correlations between serum 25(OH)D levels, BMI, and geographic area were calculated. Individuals were grouped according to their serum 25-hydroxyvitamin D levels (ng/ml): severe deficiency: <5; moderate deficiency: 5–10.9; mild deficiency: 11–20.9; insufficiency: 21–29.9 and sufficiency: 30–100.

**Results:**

We found 80.4% of children had serum 25(OH)D deficiency, with 1.7% severe, 24.6% moderate, and 54.1% mild. In the three cities, the percentage of serum 25(OH)D deficit was increased when comparing overweight or obesity with a healthy weight. Additionally, an interaction effect was observed between geographic area, nutritional status, and serum 25(OH)D levels using the factorial ANOVA test (*p* = 0.038). In Antofagasta, there were more overweight children and also a higher percentage of children with VitD deficiency (<30 ng/ml) compared to Santiago or Concepción.

**Conclusion:**

This study revealed a high prevalence of serum 25(OH)D deficiency in children between 4 and 14 years old in Chile (80.4%) during May–September 2018. Obese and overweight children had the highest prevalence of serum 25(OH)D deficiency.

## Introduction

Vitamin D (VitD) is essential for developing and maintaining a healthy skeleton, and it has been linked to reduced risk for acute and chronic illnesses [1]. VitD deficiency is associated with obesity, cardiovascular disease, insulin resistance, beta-cell dysfunction, autoimmune diseases, and cancer [2]. Several factors can influence VitD status, including sunlight, diet, and dietary VitD supplements. Besides, lifestyle factors such as for overweight, obesity, and sedentarism also influence VitD status [3].

In Chile, a limited number of studies conducted on healthy children have shown evidence of the impact of nutritional status and sunlight exposure on VitD levels [4].

Childhood obesity is a public health problem in Chile. The latest data published by the OECD show that 74% of the adult Chilean population are overweight or obese. Furthermore, the FAO report indicates that Chile has one of the highest rates of childhood overweight (9.3%) in Latin America and the Caribbean [5].

The objective of this study was to analyze serum 25(OH)D levels in a representative sample of children between 4 and 14 years of age located in the Santiago, Antofagasta, and Concepción.

## Materials and methods

The study was conducted from May to September 2018 with 1134 children aged 4–14 years from different latitudes and UV index (UVI): Antofagasta (north of Chile: 23.65°; 6–7 UVI), Santiago (central zone: Santiago 33.70°; 4–6 UVI), and Concepción (southern region: Concepción 36.73°; 3–5 UVI). Study participants were recruited independently of nutritional status according to gender, age group, and socioeconomic status. Children were grouped according to their serum 25(OH)D levels [6].

Anthropometric measurements were made with children in light clothing, weight, and height were measured with a portable stadiometer (SECA 813/213). The nutritional status classification was carried out according to the WHO standards for the age and gender of the children. Children with BMI/Age < +1 DS were classified as healthy weight, those with BMI/Age between +1 SD and +2 SD were classified as overweight, and those with BMI/Age ≥ +2 SD as obese. Children with chronic diseases, such as diabetes, cancer, or chronic kidney disease, were excluded. Informed consent was obtained from all subjects. The study protocol was reviewed and approved by the Ethics Committee of the University of Chile.

### Biochemical measurements

Serum 25(OH)D was measured in triplicate using an Enzyme-Linked Immunosorbent Assay kit (ELISA, DiaSource, Belgium). Sensitivity of 2 ng/ml with an intra-assay variation coefficient of 5.1% and an inter-assay variation coefficient of 5.9%.

### Statistical analysis

Results are presented as percentages (%) and means ± SD. All calculations were performed using the software package SPSS version 15.0.1 (SPSS Inc, Chicago, IL). Shapiro–Wilk normality test was performed to determine the distribution of the variables. Pearson correlation test, factorial ANOVA, one-way ANOVA, and Tukey posthoc analysis were used. In all cases, a value of *p* < 0.05 was considered significant.

## Results

Antofagasta’s children presented both the highest weight, height, waist circumference, BMI, and height/age ratio relative to the other cities evaluated in this study. We showed a high level of serum 25(OH)D deficiency in the three cities. The serum 25(OH)D levels were significantly higher in Antofagasta compared to Concepción (Table 1). In Antofagasta were higher levels of 25 (OH) D in boys when compared with girls (*p* = 0.041). In the three cities, the serum 25(OH)D deficit was increased when comparing overweight or obesity with a healthy weight. An interaction effect was observed between geographic area, nutritional status, and VitD levels using the factorial ANOVA test (*p* = 0.038). In Antofagasta, there were more overweight children and also a higher percentage of children with VitD deficiency (<30 ng/ml) compared to Santiago or Concepción.

**Table 1:** Anthropometric, measurements and daily consumption in children by cities (mean ± SD).

	Antofagasta (*n* = 354)	Santiago (*n* = 396)	Concepción (*n* = 384)	*p*-value
Age (years)	10.1 ± 2.7^a^	9.5 ± 2.7^b^	10.0 ± 2.8^a,b^	<0.01
Weight (Kg)	46. ± 17.0^a^	40.1 ± 15.7^b^	42.9 ± 15.9^b^	<0.001
Height (cm)	143.0 ± 16.7^a^	139.36 ± 17.5^b^	142.6 ± 17.2^a,b^	<0.01
Waist circumference (cm)	69.9 ± 12.0^a^	64.2 ± 10.6^b^	68.2 ± 11.4^a^	<0.001
BMI (Kg/m^**2**^)	21.7 ± 4.7^a^	19.8 ± 3.8^b^	20.3 ± 4.1^b^	<0.001
zBMI/Age	1.2 ± 1.1	1.1 ± 1.1	1.05 ± 1.1	0.320
zHeight/Age	0.50 ± 1.1^a^	0.29 ± 1.1^b^	0.11 ± 0.9^c^	<0.001
Dairy consumption (days/wk)	5.3 ± 2.1^a^	6.1 ± 1.8^b^	5.8 ± 1.9^b^	<0.001
Dairy consumption (portions/day)	1.5 ± 0.7^a^	1.9 ± 0.8^b^	1.6 ± 0.8^a^	<0.001
Fish consumption (days/wk)	1.0 ± 0.9	1.1 ± 1.0	1.1 ± 1.0	<0.05
25(OH)D (ng/ml)^c^	15.1 ± 6.5^a^	13.9 ± 7.1^a,b^	13.8 ± 5.2^b^	<0.04

One-way ANOVA and Tukey post hoc.

^a,b^Mean values within a row with unlike superscript letters were significantly different.

^c^Gender differences only in Antofagasta.

**Fig. 1:**
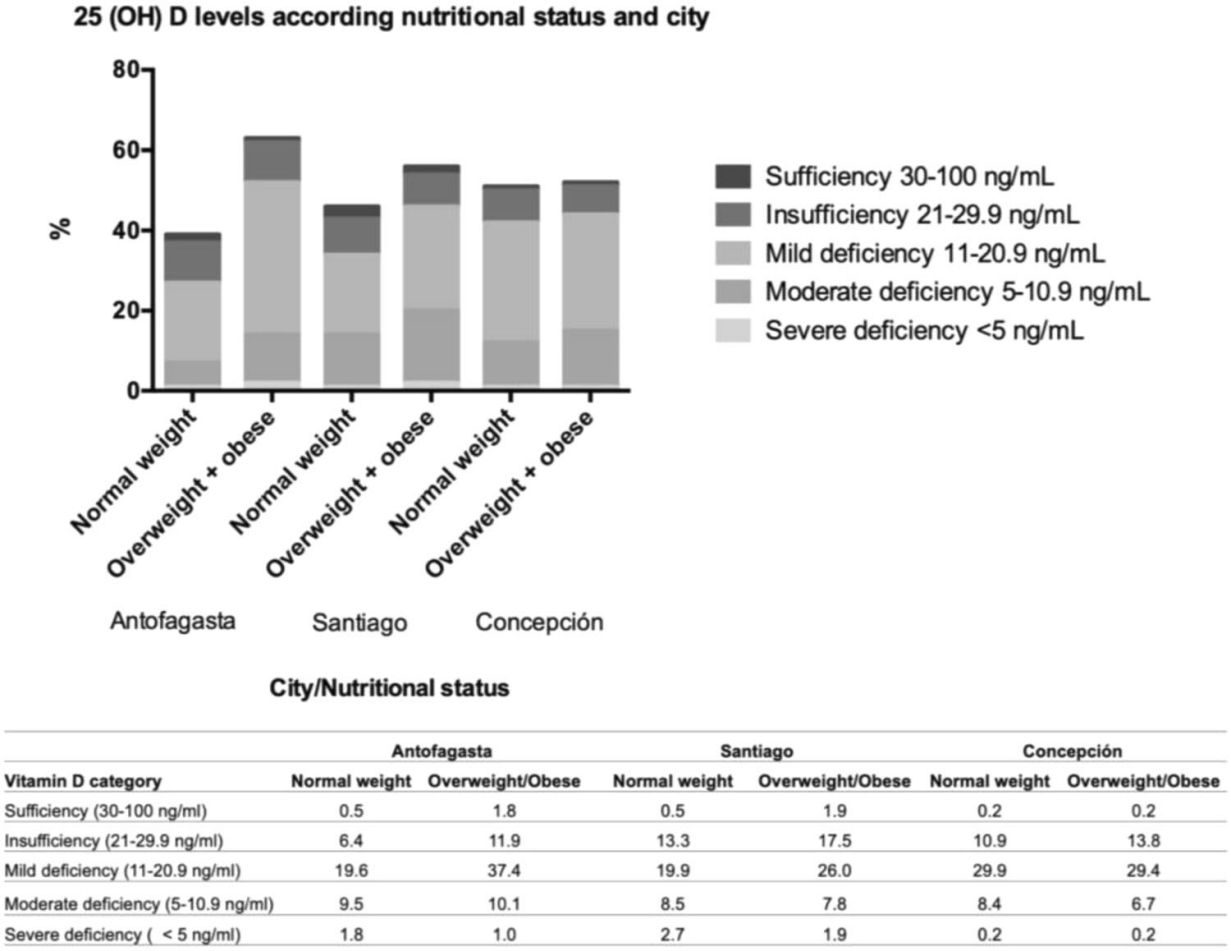
Serum 25(OH)D levels by nutritional status in Antofagasta, Santiago, and Concepcion. Factorial ANOVA (*p < 0.05). *p* City = 0.744, *p* Nutritional status = 0.278, *p* Vitamin D level = 0.008*, *p* Interaction = 0.038*. According to WHO standards, Nutritional status includes children with BMI/Age < +1 DS classified as normal weight, with BMI/Age between +1 SD and +2 SD classified as overweight, and those with BMI/Age ≥ +2 SD as obese. VitD levels are influenced (jointly, not as independent factors) by the nutritional status of the children and the geographical location where these children live.

Only 22.4% in Antofagasta, 20.9% in Santiago, and 15.5% in Concepción reached levels of serum 25(OH)D > 20 ng/ml (Fig. 1). Finally, regression analysis between the variables gender, age, nutritional status, city, and vitamin D status showed that vitamin D is lower at older ages in Santiago and Concepción compared to Antofagasta (*p* < 0.0001).

## Discussion

VitD deficiency was higher in the presence of overweight and obesity. In 2016, Cediel et al. [4] reported deficiencies in 31.9% of girls and 50.2% of boys, who associated this deficiency with a higher percentage of adiposity. The prevalence of serum 25(OH)D deficiency in school children in Colombia reaches 12% and in Mexico is 24% of preschoolers and 10% of school children. In Spain, there are better regional data: 5% deficiency in Madrid (children between 9 and 13 years old), 45.2% deficiency in Cádiz (a city with high sun exposure) in children between 10 and 14 years old [7, 8]. In Navarra, children aged 9.1–13.9 years showed a significantly higher serum 25(OH)D deficiency in obese compared to normal-weight children (31% versus 14%). Our data show even more dramatic serum 25(OH)D deficiency than data from Spain or countries with Hispanic heritage [9].

Regardless of geographic location, the magnitude of the deficiency in the analyzed areas has powerfully attracted our attention since they differ in sunlight exposure. During May to September 2018, cloudiness was: Santiago 47–58% (7–23 °C), Antofagasta 73–84% (13–24 °C), and Concepción 40–60% (6–16 °C). Despite this variation, we believe that the high prevalence of overweight/obesity in these regions is the primary condition for VitD sequestration or dilution. In the southern areas of Chile (Punta Arenas), a high prevalence of obesity has been described in school children groups, accompanied by severe VitD deficiencies. Obesity and lack of exposure to sunlight are likely to cause these severe deficiencies [10]. While our study is not without limitations, we believe that sunscreen use, milk consumption, and physical activity are less important than the high prevalence of overweight and obesity in the Chilean population, directly impacting the magnitude of vitamin D deficiency. Serum 25(OH)D deficiency could be overestimated, in part because the measurement was carried out in the coldest months (Antofagasta should be considered an exception). In conclusion, our results show a significant deficiency of VitD in Chilean children and adolescents. The decrease in serum VitD is correlated with a higher rate of overweight/obesity, demonstrating that both problems are closely linked to evident long-term adverse effects.

## Data Availability

The datasets used and/or analyzed during the current study are available from the corresponding author on reasonable request.
